# Teacher Training and Engagement in Health Promotion Mediates Health Behavior Outcomes

**DOI:** 10.3390/ijerph19053128

**Published:** 2022-03-07

**Authors:** Maha Nubani Husseini, Donna R. Zwas, Milka Donchin

**Affiliations:** 1 Faculty of Public Health, Al-Quds University, Abu Dis 22100, Palestine; 2 Linda Joy Pollin Cardiovascular Wellness Center for Women, Division of Cardiology, Hadassah University Medical Center, Jerusalem 9574425, Israel; donnaz1818@gmail.com; 3 Braun School of Public Health, Hadassah & The Hebrew University-Hadassah Medical School, Jerusalem 9574425, Israel; milka@hadassah.org.il

**Keywords:** mediation–moderation, teachers’ training, teachers’ engagement, school health promotion program, school setting

## Abstract

School-based health promotion interventions have been shown to lead to measurable changes in the nutrition and physical activity behaviors. This study examines whether the impact of an intervention program on students’ healthy eating and physical activity was mediated by teacher training and engagement in health promotion. The trial was conducted in three phases: needs assessment of the baseline survey of teachers, mothers’ and children; intervention among seven randomly selected schools that included teacher training in healthy eating and physical activity; and a post-intervention evaluation survey. The SPSS PROCESS for Hayes (Model8) was used to determine moderation and mediation effects. The difference in difference (DID) was calculated for the three main outcomes of the study: eating breakfast daily (DID = 17.5%, *p* < 0.001); consuming the recommended servings of F&V (DID = 29.4%, *p* < 0.001); and being physically active for at least 5 days/week (DID = 45.2%, *p* < 0.001). Schoolchildren’s eating breakfast daily was mediated by their teachers’ training in nutrition (β = 0.424, *p* = 0.002), teachers’ engagement (β = 0.167, *p* = 0.036), and mothers preparing breakfast (β = 1.309, *p* < 0.001). Schoolchildren’s consumption of F&V was mediated by teachers’ engagement (β = 0.427, *p* = 0.001) and knowing the recommended F&V servings (β = 0.485, *p* < 0.001). Schoolchildren’s physical activity was mediated by their teachers’ training in physical activity (β = 0.420, *p* = 0.020) and teachers’ engagement (β = 0.655, *p* < 0.001). Health behavior changes in the school setting including improvements in eating breakfast, consuming the recommended F&V and physical activity was mediated by teacher training and engagement. Effective teacher training leading to teacher engagement is warranted in the design of health-promotion interventions in the school setting.

## 1. Introduction

The school setting is an optimal environment for health promotion in children. As children spend most of their day in school, the school framework can powerfully influence eating habits [1] and physical activity [2], and provide a safe and supportive environment that enables children to learn and implement healthy practices [3,4]. Schools can play a critical role in the prevention of overweight and obesity in children. Additionally, utilizing existing social settings such as a school can facilitate dissemination of health interventions [5]. Although many successful school-based interventions have been described, there is limited understanding of the underlying mechanisms of healthy eating and physical activity behavior changes in school-based interventions [6].

Teachers are considered both gatekeepers in implementing intervention programs [7] and stakeholders in creating a sense of ownership towards the program [8]. They are fundamental partners in developing and modifying classroom practices, policies and strategies [7] within the school setting. Students in elementary schools consider teachers to be role models as well as educators [9,10]. This dual role increases teachers’ impact on students’ behaviors. As role models, teachers’ consumption of healthy food in the classroom provides an effective opportunity for teaching children to make healthy choices. Fostering teachers’ commitment, interest, and competence in contributing to the program is therefore essential for a successful health promotion program [9,10]. A recent review of teachers’ training programs suggested that the teacher training component of school-based physical activity interventions is under-reported and under-studied, and the role and impact of teacher training is insufficiently understood [11].

A mediator can be defined as a necessary intervening variable, needed to complete the pathway from an intervention to the targeted behavioral outcome [12]. A mediation analysis is considered the tool for assessing the mediators of an intervention effect. It also gives a better understanding of the different components of an intervention and determines their effectiveness [13]. The current literature suggests that the understanding, conduction and presentation of the mediation analysis present a challenge [13]. However, mediation analysis in the assessment of intervention trials has been proven to be useful when conducted properly, as it statistically identifies an intermediate variable that relates an independent variable to an outcome [14]. Furthermore, randomized controlled trials are regarded as the ‘gold standard’ for healthy eating and physical activity interventions, thus providing a valuable opportunity to identify the mediators of behavioral changes [15]. 

This study tests the hypothesis that the impact of an intervention program on students’ healthy eating and physical activity is mediated by teachers’ training and engagement in health promotion.

## 2. Materials and Methods

### 2.1. Study Design and Participants

A randomized controlled intervention program trial was carried out in 14 girls’ elementary schools in East Jerusalem, with random allocation stratified by the four groups of schools that operate in East Jerusalem (schools are operated by the Palestinian Authority (P.A.), the Jerusalem Municipality, the United Nations Relief and Works Agency (UNRWA) or are privately owned). The study design included independent cross-sectional samples of female schoolchildren in the 4th and 5th grades and their mothers, comparing health behaviors in the samples from the intervention and the control group, before and after the 2-academic-year intervention.

The students were the primary target population for the intervention, whereas the secondary target populations were the teachers and the mothers at these schools.

Theoretical framework: The study process was designed using the socio-ecological model, which identifies the position of the individual within a larger social system and describes the individuals’ and environments’ characteristics that affect the health outcomes [16]. The intervention was carried out in three stages: needs assessment, intervention and evaluation (described in detail elsewhere [17]). Stage one: Needs assessment—a semi-structured interview was conducted with all 14 school principals, and a structured self-administered questionnaire was used with all 373 teachers. School inspection tours were completed to assess the school’s health environment. A random sample of 4th- and 5th-grade classes was selected, in which all mothers and their daughters were asked to answer a self-administered questionnaire which was based on a validated translation of the Health Behavior of School Children questionnaire (HBSC) [18]. The height and weight of the children were measured during this stage. These measurements provided the baseline for planning the intervention.

Stage two: Intervention—Schools were stratified by administering body and randomized into intervention and control groups. Data collected at each school were collated and analyzed and presented via power point and written report to representative from each school, including the principals, their deputies and teachers. Data from that school were compared to the composite study data. This was followed by participatory planning and implementation of the intervention in each of the 7 schools based on their particular needs and assets. The health promotion program was designed, implemented and administered by a steering committee in each of the schools, consisting of teachers, mothers, and children. In each school, a teacher was appointed as the program coordinator and headed the health steering committee. The health steering committee consisted of representatives of teachers, mothers, schoolchildren and the owner of the canteen (8–10 individuals). Although each school planned their activities according to their specific needs and assets, ideas were shared through the researcher and the school health coordinator’s visits to other intervention schools. Teachers in each intervention school underwent training which is detailed below. The program activities were monitored by the researcher through regular visits twice a month. The implementation team met every four to six weeks to review the progress.

Stage three: the same assessment questionnaires for both intervention and control schools among a different sample of children from grades 4 and 5, their mothers, and all the teachers. The study has been described in detail elsewhere [19].

### 2.2. Intervention Content

The 18-month-intervention included seven educational workshops for mothers held in each of the schools (120–150 min long), focusing on the importance of healthy eating (Mediterranean diet pattern) and physical activity. The schools were encouraged to create a supportive health environment and health-promoting policies, such as decorating with health messages at the classrooms and staircases; playgrounds were decorated to encourage physical activity and the canteen offerings were changed, integrating health messages into morning announcements and health content into the curriculum. The teacher capacity building program included intensive training of all teachers through a five-session training program that was held during the first year of the intervention. Each session was 120–150 min long and was presented by professionals in the fields of nutrition, physical education and health promotion. The teachers’ training program was conducted in each school separately, by the same professional team. Teachers’ sessions incorporated methods that promote healthy eating and physical activity in the school setting, in addition to building a school health promotion program. The curriculum for the training sessions was in accordance with the research goals and objectives and based on the needs assessment.

The health promotion programs designed by the steering committees at each school included numerous components designed to implement changes in school policies and the environment to promote healthy nutrition and physical activity. Examples include a checklist to record the schoolchildren’s daily habits of eating breakfast before school, drinking milk, and bringing healthy lunches. Weekly/monthly rewards were offered to the schoolchildren based on healthy habits; these rewards included healthy snacks or school stationery. The schoolchildren were instructed to eat in class under the supervision of their teachers who in return had to set an example by consuming healthy breakfast themselves, followed by an active break in school yards that had been set up with traditional games. The intervention activities encompassed the whole school setting and not only the sampled 4th and 5th graders.

### 2.3. Measures

The primary outcomes of the study, eating breakfast, fruit and vegetable consumption, and physical activity on the part of the schoolchildren were measured pre- and post-intervention using a validated Arabic translation of the Health Behavior of School Children questionnaire (HBSC) [18]. Eating daily breakfast was coded as yes if the child answered “always”, and no if the child answered “sometimes and never”. To ensure the validity of the schoolchildren’s responses in the questionnaire, their answers regarding consumption of daily breakfast were compared to the follow-up checklists obtained from one of the intervention schools, in which also recorded daily breakfast consumption was recorded during the last three months of the intervention.

The responses to the questions regarding average daily consumption and servings of fruits and vegetables were coded into two categories of less than five or greater than or equal to five servings per day. This was calculated by multiplying the number of days per week the schoolchildren consume fruits by the number of daily servings divided by seven, the same was carried out to calculate the intake of vegetables. To assess their physical activity habits, schoolchildren responded to the following question “over the past 7 days, on how many days were you active in sport at least one hour per day”, which was categorized into two categories: less than five or greater than or equal to five days a week of physical activity. Knowing the recommended daily servings of vegetables & fruits; was converted to 3 categorical variables: (1) do not know, (2) might know and (3) know.

Teachers’ level of engagement in or intention to become engaged in health promotion at school was measured as an ordinal variable based on the “Transtheoretical Model”, using the “stages of change” technique [20]. This was assessed in the pre- and post-intervention questionnaires, teachers were asked to choose statements that best described their stage of readiness with respect to engagement in health promotion at their school; then question was categorized into engaged, intend to be engaged and not engaged. For further analysis, those who responded “intend to be engaged” were coded as not engaged, leading to a dichotomous variable (engaged vs. not engaged)

*Teacher’s training in health promotion was assessed using 9 thematic items assessing their training in healthful eating, physical activity. A Likert scale was used for each item, ranging from no training (1 point) to highly trained (5 points). Cronbach’s alpha of the responses to this scale was 0.87, suggesting scale reliability. Score were summed and averaged by the number of responses, leading to an average score of training ranging from 1 (no training in all subjects) to 5 (highly trained in all subjects). This average was categorized into (1) low level of training (1–2 points), (2) moderate level of training (3 points), and (3) high level of training (4–5 points).*

### 2.4. Statistical Analyses

A time variable was defined as the pre- versus post-intervention sub-samples, where the pre- and post-intervention sub-samples were independent of each other, yet were homogeneous in the intervention schedule. Thus, time was set as an independent effect.

A logistic regression was designed to calculate the probability of engagement of teachers in health promotion as a response to a variety of independent variables, where the dependent variable was the binary decision to engage versus not to. The outcomes of the logistic regression were the probabilities of engaging in the health promotion program [21].

A stepwise likelihood ratio logistic regression model was built for identifying independent explanatory factors (i.e., school types of ownership, religion, mothers’ education level and employment status, crowding index, birth order, and teacher’s engagement in health promotion) on schoolchildren eating breakfast daily, engaging in physical activity and being overweight and obese, which were all categorized into a dichotomous scale. Specifically, this stepwise regression was hierarchical in the sense that research factors, healthy eating and physical training, were entered last, to assess their additional effect to the explanatory power of the model by means of R^2^ change and F.

**Figure 1:**
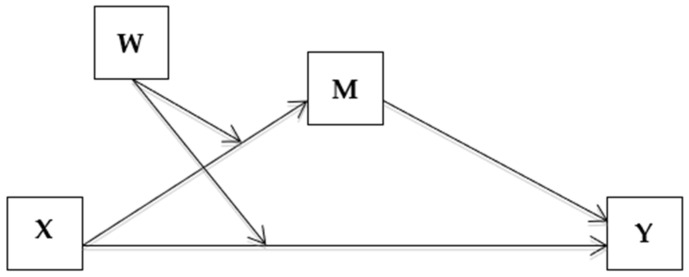
Conceptual Model 8.

The moderated mediation model was used in order to examine the research hypothesis that the impact of the intervention program (time: before versus after) on students’ healthy eating and physical activity will be mediated by teachers’ engagement in health promotion, subject to the moderating effect of group affiliation. Model 8 was used from Hayes’s PROCESS for SPSS [22] (Figure 1), which describes the moderating effects (AKA interaction) of the intervention group versus the control on two regression pathways: the pathway from X (time; pre- versus post-intervention, see measure definition above) to the mediator M (teachers’ training type), and the pathway from X to Y (the dependent variables—schoolchildren eating breakfast, consuming recommended quantity of fruits and vegetables and physical activity) (W to M; W to Y). That is, the effect of the independent variable time on the dependent variables may be mediated by another variable termed as “mediator” (mothers’ behavior, teachers’ engagement in health promotion and training, and environmental changes), while the “moderator”, the intervention group, would lead to differences in the degree of mediation, thus in the setting of moderated mediation, for each intervention group we expected the mediation effect to be different. Note that according to Figure 1, the group moderation was set to affect both the pathway from the independent time to the mediators, and from time to the dependent variable. Thus, the indirect effect from X to Y through M may vary by group. The “covariates” school type and crowding index (as a proxy to social class) were controlled for as they might be confounders.

To calculate the net effect of the intervention for each of the main outcome (dependent) variables, the difference in differences (DID) of post- minus pre-intervention among the intervention population was calculated in relation to the post- minus pre-intervention among the control population using WinPepi [23], where the prevalence and standard error (SE) were calculated for each of the intervention and control groups at the pre and post intervention, and then the difference between their means was calculated. Simply put, the DID is an odds ratio which reflects the net change in the intervention group over time when taking into consideration the changes that occurred in the control group over the same time. This enables an accounting for factors other than the treatment that may influence the outcome over time, and helps overcome selection bias. To verify the answer, logistic regression was used, which gave identical results.

## 3. Results

**Figure 2:**
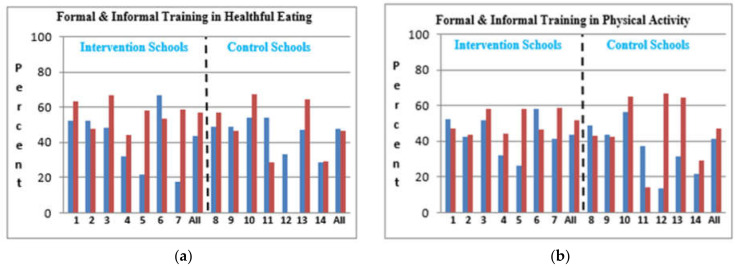
Teachers’ characteristics (%) in each school for intervention vs. control at baseline and post-intervention—blue is pre intervention and red is post intervention.

897 schoolchildren participated in the pre-intervention study, while the sample at the post-intervention assessment included 866 schoolchildren (mean age = 11.02 SD ± 0.73, mean family size was 7.1). Table 1 presents the socio-demographic characteristics of the study population by school ownership. About 94% were Muslims, while 6% were Christians, all attending private schools. The mean family size was 7.1; Schoolchildren from Municipality, P.A and UNRWA had more siblings compared to those in private schools. Schoolchildren from Municipality and UNRWA schools reported a higher crowding index (residents per room) compared to those attending PA and private Schools. Eighty one percent of the mothers did not work and 20% had a diploma or higher education. Further detailed description of the participants has been reported previously [17]. The post-intervention assessment showed improvements in the intervention schools regarding the formal and informal training teachers received in healthy eating and physical activity (Figure 2).

The post intervention assessment indicated a significant net difference in outcomes between the intervention and control schools. For eating breakfast daily, the DID was 17.5% (*p* < 0.001); for consuming the recommended servings of fruits and vegetables, it was 29.4% (*p* < 0.001); and for being physically active for at least 5 days per week, it was 45.2% (*p* < 0.001). The odds ratio for finding a difference in the intervention group compared to the control group for eating breakfast was 1.82 (CI 1.02–3.25, *p* = 0.042); for eating the recommended servings of fruits and vegetables it was 1.98 (CI 1.03–3.81, *p* = 0.040); and for physical activity, it was 6.6 (CI 3.16–11.56, *p* < 0.001), when controlled for baseline predictors. The post-intervention assessment showed that the odds ratio of an increase in overweight and obesity in the intervention group was 0.73 (OR= 0.73, 95% CI 0.57–0.94, *p* = 0.016).

### 3.1. Mediators and Moderators of Teachers’ Engagement

**Figure 3:**
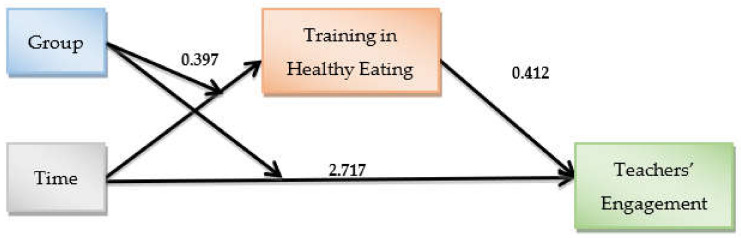
Empirical model results for teachers’ engagement and their training in healthy eating.

Teacher training in healthy eating and physical activity was evaluated and tested separately to determine the effect on teachers’ engagement in the school health promotion program (Figure 3). The school’s type of ownership was used as a covariate in both models—that is, an additional control beyond the model elements. The coefficient for the interaction between time and group in the model of training in healthy eating was 0.397, which was statistically different from zero (*p* < 0.001). The effect of training in healthy eating on teachers’ engagement, controlled for all other variables in the regression was 0.412, and it was statistically different from zero (*p* < 0.001). This indicates that the higher the self-assessment of training score the teachers received, the more they became engaged. The model also provides the significant indirect effect of the interaction (time X group) on teachers’ engagement through self-assessment of teacher training in healthy eating, which is the product of the above-mentioned coefficients (β = 0.164, SEboot = 0.066, 95% CIboot = 0.0635–0.3205). This conditional indirect effect was significant for the intervention group (β = 0.204, SEboot = 0.066, 95% CIboot = 0.090–0.3482) and not for the control group (β = 0.040, SEboot = 0.038, 95% CIboot = −0.204–0.640), where the 95% bootstrap confidence interval for these indirect effects was wholly above zero in the intervention and not in the control.

**Figure 4:**
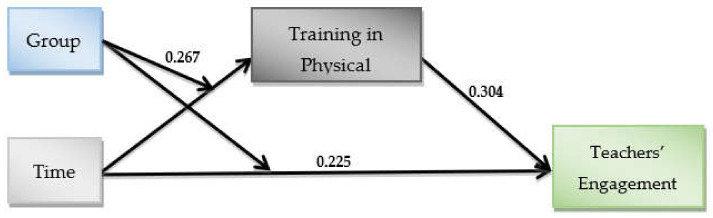
Empirical model for teachers’ engagement & their training in physical activity.

There was also a statistically significant indirect effect of the interaction (time X group) on teachers’ engagement through teachers’ self-assessment of training in physical activity (β = 0.081, SEboot = 0.048, 95% CIboot = 0.013–0.205) (Figure 4). This was detected in the intervention group and not in the control. The conditional indirect effect for the intervention group (β = 0.111, SEboot = 0.048, 95% CIboot = 0.033–0.223) was higher than that in the control group (β = 0.030, SEboot = 0.028, 95% CIboot = −0.011–0.100), where the 95% bootstrap confidence interval for these indirect effects was wholly above zero in the intervention and not in the control.

### 3.2. Predictors/Mediators of Schoolchildren’s Health Behavior

Table 2 shows the coefficients and standard error of the mediation of teachers’ engagement mediation on schoolchildren eating breakfast daily, predictors and covariates. There was a statistically significant indirect effect of the interaction (time X group) on schoolchildren’s consumption of daily breakfast through their teachers’ engagement (β = 0.092, SEboot = 0.045, 95% CIboot = 0.052–0.186). The conditional indirect effect for the intervention group (β = 0.130, SEboot = 0.062, 95% CIboot = 0.006–0.249) was higher than that of the control group (β = 0.039, SEboot = 0.019, 95% CIboot = 0.003–0.079), where the 95% bootstrap confidence interval for these indirect effects did not include zero in the intervention and the control (Table 2). The crowding index was used as a covariate. The table also shows similar results for mothers’ preparing breakfast and teacher’s training.

There was also a statistically significant indirect effect of the interaction (time X group) on schoolchildren consuming the recommended number of daily servings of fruits and vegetables via teachers’ engagement (β = 0.250, SEboot = 0.065, 95% CIboot = 0.145–0.396). The conditional indirect effect for the intervention group (β = 0.336, SEboot = 0.080, 95% CIboot = 0.185–0.507) was higher than that of the control group (β = 0.086, SEboot = 0.025, 95% CIboot= 0.043–0.146), where the 95% bootstrap confidence intervals for these indirect effects were wholly above zero in the intervention and the control. Mothers’ level of education was used as a covariate since this was associated with the children’s consuming the recommended daily servings of fruits and vegetables at baseline (Table 3). In addition to teachers’ engagement, the table also presents the mediation analysis for schoolchildren’s knowledge on the outcome of schoolchildren consuming recommended servings of fruits and vegetables.

There was also a statistically significant indirect effect of the interaction (time X group) on physical activity in schoolchildren more than 5 times a week through teachers’ engagement in HP (β = 0.361, SEboot = 0.060, 95% CIboot = 0.257–0.496). This was detected in the intervention group and not in the control. The conditional indirect effect for the intervention group (β = 0.512, SEboot = 0.074, 95% CIboot = 0.381–0.678) was higher than that in the control group (β = 0.151, SEboot = 0.032, 95% CIboot= 0.097–0.229), where the 95% bootstrap confidence interval for these indirect effects was wholly above zero in both the intervention and the control. The crowding index was used as a covariate since this variable was a predictor for being physically active for more than five times a week at the baseline (Table 4). Living in a crowding index of more than two persons per room decreased the probability of being physically active by 60% (95% CI 0.20–0.74). The table also shows the coefficients and standard error of teachers’ training on schoolchildren being physically active (≥5 times/week) predictors and covariates.

## 4. Discussion

This study found that positive outcomes in a school-based health promotion program were mediated by the teachers’ training and engagement in health promotion. Specifically, there was a significant impact of training on teacher engagement in school HP, and teacher engagement affected student behavior regarding healthy eating and physical activity.

In previous studies on nutrition and physical activity behavior changes in children, mediation analysis added to the understanding of the mechanism of the changes [24], as it permits the researcher to identify which of the intervention components were directly or indirectly associated with the behavior changes [25]. Another study on self-efficacy and behavioral capability also showed the mediating effect of teachers on the behavioral changes among children [26]. Such evidence creates a guide for the development, implementation, evaluation and modification of future interventions and enhances the understanding of how the results were achieved [13]. This study is the first, to our knowledge, that quantifies the mediating role of teacher training and teacher engagement in school-based health promotion targeting nutrition behaviors and physical activity.

These findings add to the descriptive literature that suggests that training of teachers and coordinators is a major component in the success of school-based health promotion interventions [27,28] and interventions specifically targeting physical activity [29,30]. Such training could effectively motivate the teachers in implementing the behavioral changes curricula in the classrooms [31]. A number of cross-sectional studies found a positive association between training staff in physical education and the students’ physical activity [11]. Teacher training enables the teachers to be well-informed of the changes that will occur due to the intervention and to be able to fulfill their intended roles in the implementation [32]. As has been recognized by many studies, training can enrich teacher knowledge, attitudes, health behaviors, and teaching skills towards the training subject [33,34].

Health promotion training also influences teachers’ health self-efficacy [27]. Other intervention studies found that teacher training in health promotion is positively associated with increased efficacy in implementing new health curriculum compared with the teachers who do not receive this training [35]. As part of the teacher capacity building in this program, all teachers in the school were given the opportunity to received intensive training in healthy eating and physical activity. This differs from other similar programs that trained only 50% of the teachers [36] or just one teacher in each school [37].

The teachers training program in this intervention sought to increase teachers’ knowledge and attitude towards healthy eating and physical activity, and targeted integration of these topics into varied subjects of teaching. For example, the mathematics teacher started giving examples using fruit and vegetables, the geography teacher used examples of where/how certain foods are grown in the country, etc. Teachers have the ability to integrate the elements of wellness and health into their educational curriculum, which can greatly enhance the effectiveness of a program’s nutrition education [38]. It is recommended that integration of health topics into the curriculum be comprehensive rather than simply a one-time class topic [38].

This study demonstrated that the training program increased the teachers’ engagement in health promotion. In one school, teachers decided to have a competition between them to show which has the best students. Other smaller studies have also identified the role of teacher engagement in forwarding health promotion [28,39] programs, but this remains an understudied element in school-based health promotion [11]. In this study, the teachers set out a role model for their schoolchildren by eating healthy breakfast in class, with their schoolchildren daily. When the schoolchildren started eating in class, they viewed their teachers’ eating habits and were encouraged by their teachers to consume healthier food products and to decrease their intake of salty snacks, chocolates, and sweetened juices.

### Limitations

This program was implemented only in girls’ elementary schools in East Jerusalem, and effectiveness among boys’ schools or mixed classrooms requires further testing. Similarly, the majority of teachers in this study were female. The questionnaire was self-administered by children in grades 4 and 5, which could have influenced its validity and reliability. Studies show that results from self-administered questionnaires tend to minimize social desirability bias compared to interviewer administration [40]. The investigator was present during data collection, but to minimize this bias, students were told that there were no right or wrong answers. As with all moderation-mediation models, unidentified confounders may affect the causal interpretation of the findings. The randomized nature of the study and varied schools should minimize these effects. The Hayes’s PROCESS for SPSS technique is also limited by the necessity of discretizing the variables, for example engagement, which may lead to loss of information on more subtle differences.

## 5. Conclusions

This study examined the mediation effect of teachers’ training and engagement on school-based interventions. Our findings confirm the hypothesis that the positive impact of a school-based health promotion intervention targeting students’ healthy eating and physical activity behaviors was mediated by teachers’ training and engagement in health promotion. To summarize, this study suggests that teacher training and increased engagement are key features that will enhance the efficacy of interventions targeting healthy eating and physical activity. Teachers should be trained prior to program implementation in accordance with the program content in order to increase their engagement in health promotion.

Moreover, mechanisms to promote teacher engagement should be further explored, and teacher training and engagement should be emphasized in school-based health promotion interventions. Implementation of school health promotion education programs with teachers and school staff, including methods to incorporate health education into the standard curriculum, as well as support in planning and implementing activities to promote healthy behaviors is likely to increase the likelihood of effective school-based interventions.

## Figures and Tables

**Table 1:** Schoolchildren’s socio-demographic characteristics of the study population in each school ownership at both baseline and post-intervention.

	Baseline					Post-Intervention									
						Control					Intervention				
School Type	JM	PA	UNRWA	Private	Total	JM	PA	UNRWA	Private	Total	JM	PA	UNRWA	Private	Total
Number of Schoolchildren	400	236	136	125	897	191	122	65	42	420	192	116	71	67	446
Schoolchildren’s Grade (%)															
4th Grade	49.8	50.0	49.3	51.2	49.9	54.5	52.5	46.2	61.9	53.3	45.3	49.1	50.7	47.8	47.5
5th Grade	50.2	50.0	50.7	48.8	50.1	45.5	47.5	53.8	38.1	46.7	54.7	50.9	49.3	52.2	52.5
Schoolchildren’s Age															
Mean	11.02	11.00	11.10	10.98	11.02	11.01	11.05	10.86	11.14	11.01	11.10	10.86	11.05	11.01	11.02
SD	0.70	0.78	0.85	0.71	0.75	0.73	0.70	0.73	0.81	0.73	0.69	0.63	0.71	0.72	0.69
Maximum	13	14	14	13	14	13	12	13	13	13	13	12	13	12	13
Minimum	10	9	10	9	9	10	10	10	10	10	10	10	10	10	10
Birth Order (%)															
1	19.2	19.1	16.9	29.6	20.6 ***	22.0	17.2	10.8	23.8	19.0	19.3	20.7	11.3	25.8	20.9
2–3	39.0	30.9	37.5	51.2	38.4	45.5	39.3	44.6	52.4	44.3	44.8	44.8	43.7	32.8	42.8
4	15.6	16.1	15.4	9.6	15.2	11.0	18.0	21.5	16.7	15.2	13.0	11.2	21.1	13.4	13.9
5+	24.8	33.9	30.1	9.6	25.9	21.5	25.4	23.1	7.1	21.4	22.9	23.3	23.9	17.9	22.4
Sibling (%)															
0–2	14.5	6.8	5.9	43.2	15.2 ***	9.9	9.8	1.5	38.1	11.4 ***	15.6	9.5	15.5	26.9	15.7 ***
3–4	44.8	42.4	38.2	46.4	43.4	48.2	47.5	27.7	54.8	45.5	51.0	44.0	38.0	53.7	47.5
5+	40.8	50.4	55.9	10.4	41.4	41.9	42.6	70.8	7.1	43.1	33.3	46.6	46.5	19.4	36.8
Crowding Index (%)															
Up to 1	9.0	6.4	8.1	17.6	9.4 ***	3.7	7.4	1.5	14.3	5.5 **	5.7	6.0	5.6	26.9	9.0 ***
1–2	54.5	66.1	51.5	62.4	58.3	53.4	50.8	61.5	69.0	55.5	55.7	51.7	54.9	64.2	55.8
>2	36.5	27.1	40.4	20.0	32.3	42.9	41.8	36.9	16.7	39.0	38.5	42.2	39.4	9.0	35.2
Religion (%)															
Muslim	100.0	100.0	100.0	59.2	94.3 ***	100.0	100.0	100.0	26.2	92.6 ***	100.0	100.0	100.0	100.0	100.0
Christian	0.0	0.0	0.0	40.8	5.7	0.0	0.0	0.0	73.8	7.4	0.0	0.0	0.0	0.0	0.0

** *p* < 0.01; *** *p* < 0.001.

**Table 2:** Coefficients and standard error of mothers’ preparing breakfast, teachers’ training and engagement mediation on schoolchildren eating daily breakfast predictors and covariates.

	Mothers’ Preparing Breakfast			Schoolchildren’s Consumption of Daily Breakfast			Teachers’ Training in Healthy Eating			Schoolchildren’s Consumption of Daily Breakfast			Teachers’ Engagement in HP			Schoolchildren’s Consumption of Daily Breakfast		
	β	SE	*p*-Value	β	SE	*p*-Value	β	SE	*p*-Value	β	SE	*p*-Value	β	SE	*p*-Value	β	SE	*p*-Value
Intercept	2.644	0.027	<0.001	−4.126	0.373	<0.001	3.378	0.042	<0.001	1.490	0.525	0.005	2.812	0.063	<0.001	−0.670	0.308	0.029
Time (pre–post-intervention)	−0.055	0.038	0.143	−0.117	0.163	0.476	0.238	0.025	<0.001	−0.226	0.149	0.129	0.231	0.042	<0.001	−0.161	0.146	0.271
Group (intervention–control)	0.000	0.037	1.00	0.117	0.159	0.461	−0.028	0.024	0.249	0.156	0.140	0.265	0.116	0.041	0.005	0.125	0.140	0.371
Interaction- time X group	0.143	0.053	0.001	0.596	0.226	0.008	0.437	0.034	<0.001	0.565	0.207	0.006	0.549	0.059	<0.001	0.649	0.204	0.001
Mothers’ preparing breakfast				1.309	0.129	<0.001												
Teachers’ training in healthy eating										0.424	0.138	0.002						
Teachers’ engagement in HP																0.167	0.080	0.036
School type of ownership							−0.143	0.008	0.001	0.424	0.138	0.002						
Crowding index							0.032	0.015	0.027	0.004	0.051	0.936	0.126	0.025	<0.001	−0.214	0.084	0.010

**Table 3:** Coefficients and standard error of teachers’ engagement and schoolchildren knowledge mediation on schoolchildren consuming recommended servings of F&V predictors and covariates.

	Teachers’ Engagement in HP			Schoolchildren Consuming Recommended Servings of F&V			Schoolchildren’s Knowledge			Schoolchildren Consuming Recommended Servings of F&V		
	β	SE	*p*-Value	β	SE	*p*-Value	β	SE	*p*-Value	β	SE	*p*-Value
Intercept	3.501	0.049	<0.001	−2.824	0.377	<0.001	1.774	0.051	<0.001	−2.205	0.250	<0.001
Time (pre–post-intervention)	0.200	0.044	<0.001	−0.383	0.174	0.028	−0.018	0.046	0.893	−0.293	0.174	0.093
Group (intervention–control)	0.092	0.043	0.037	0.116	0.157	0.463	−0.006	0.044	0.907	0.161	0.158	0.309
Interaction time X group	0.586	0.062	<0.001	1.118	0.235	<0.001	0.572	0.065	<0.001	1.110	0.235	<0.001
Teachers’ engagement				0.427	0.092	0.001						
Mothers’ level of education	−0.2180	0.021	<0.001	0.271	0.078	<0.001						
Schoolchildren’s knowledge										0.485	0.091	<0.001
Mothers’ level of education							0.022	0.022	0.319	0.167	0.076	0.028

**Table 4:** Coefficients and standard error of teachers’ training and engagement mediation on schoolchildren being physically active (≥5 times/week) predictors and covariates.

	Teachers’ Training in Physical Activity			Physical Activity of Schoolchildren (≥5 Times/wk)			Teachers’ Engagement in HP			Physical Activity of Schoolchildren (≥5 Times/wk)		
	β	SE	*p*-Value	β	SE	*p*-Value	β	SE	*p*-Value	β	SE	*p*-Value
Intercept	2.965	0.037	<0.001	−3.1604	0.060	<0.001	2.811	0.063	<0.001	−3.732	0.392	<0.001
Time (pre–post-intervention)	0.262	0.025	<0.001	−0.154	0.205	0.452	0.231	0.042	<0.001	0.170	0.202	0.399
Group (intervention–control)	0.080	0.024	0.001	0.198	0.189	0.452	0.114	0.041	0.005	0.177	0.190	0.350
Interaction time X group	0.197	0.034	<0.001	2.046	0.258	<0.001	0.551	0.059	<0.001	1.779	0.264	<0.001
Teachers’ training				0.420	0.180	0.020						
Teachers’ engagement										0.655	0.097	<0.001
Crowding index	0.073	0.014	<0.001	−0.005	0.103	0.960	0.126	0.025	<0.001	−0.090	0.104	0.386

## Data Availability

Data are available upon reasonable request.
